# A Prediction Model for Neurological Deterioration in Patients with Acute Spontaneous Intracerebral Hemorrhage

**DOI:** 10.3389/fsurg.2022.886856

**Published:** 2022-05-27

**Authors:** Daiquan Gao, Xiaojuan Zhang, Yunzhou Zhang, Rujiang Zhang, Yuanyuan Qiao

**Affiliations:** ^1^ Department of Neurology, Xuanwu Hospital Capital Medical University, Beijing, China; ^2^ Emergency Department, Affiliated Hospital of Jining Medical University, Jining, China; ^3^ Department of Internal Medicine, Ruili People’s Hospital, Ruili, China; ^4^ Intensive Care Unit, Affiliated Hospital of Jining Medical University, Jining, China

**Keywords:** spontaneous intracerebral hemorrhage, neurological deterioration, prediction model, random forest model, factors

## Abstract

**Aim:**

The aim of this study was to explore factors related to neurological deterioration (ND) after spontaneous intracerebral hemorrhage (sICH) and establish a prediction model based on random forest analysis in evaluating the risk of ND.

**Methods:**

The clinical data of 411 patients with acute sICH at the Affiliated Hospital of Jining Medical University and Xuanwu Hospital of Capital Medical University between January 2018 and December 2020 were collected. After adjusting for variables, multivariate logistic regression was performed to investigate the factors related to the ND in patients with acute ICH. Then, based on the related factors in the multivariate logistic regression and four variables that have been identified as contributing to ND in the literature, we established a random forest model. The receiver operating characteristic curve was used to evaluate the prediction performance of this model.

**Results:**

The result of multivariate logistic regression analysis indicated that time of onset to the emergency department (ED), baseline hematoma volume, serum sodium, and serum calcium were independently associated with the risk of ND. Simultaneously, the random forest model was developed and included eight predictors: serum calcium, time of onset to ED, serum sodium, baseline hematoma volume, systolic blood pressure change in 24 h, age, intraventricular hemorrhage expansion, and gender. The area under the curve value of the prediction model reached 0.795 in the training set and 0.713 in the testing set, which suggested the good predicting performance of the model.

**Conclusion:**

Some factors related to the risk of ND were explored. Additionally, a prediction model for ND of acute sICH patients was developed based on random forest analysis, and the developed model may have a good predictive value through the internal validation.

## Introduction

Spontaneous intracerebral hemorrhage (sICH) was a major cause of disability and death worldwide, accounting for 10%–15% of strokes each year (1). Its annual incidence was 10–30 per 100,000 individuals and the mortality rate was 35%–52% within 1 month (2–4). Neurological deterioration (ND) was a devastating complication after ICH (5, 6). As ICH progresses rapidly, ND develops within the first 24 or 48 h after symptom onset (7, 8). Although stroke treatment has made some progress in recent years, ND was still a common complication in the early stage of ICH and the prognosis was relatively poor (9). Therefore, it is particularly important that early detection of patients at high risk for ND and effective clinical intervention could improve patients’ outcomes.

Several studies have investigated the risk factors of ND in patients with ICH (10, 11). One study has reported that large hematoma volumes and early hematoma enlargement (HE) were influencing factors of ND (12). Apart from that, older age, intraventricular hemorrhage (IVH), and HE-related factors [elevated systolic blood pressure (SBP)] have also been implicated as risk factors of ND after ICH (7, 12, 13). The prognosis of ND in patients with acute ICH was associated with the joint action of multiple factors; therefore, the establishment of an effective prediction model has an important clinical application value for risk assessment. However, to the best of our knowledge, existing studies based on the imageology characters to construct the prediction score associated with ND in patients with acute ICH, which generally was slightly complicated and not applicable to all patients (14, 15). In recent years, the random forest model (16), as a key data mining approach in machine learning, has been widely used in the prediction model. It could identify risk predictors by leveraging large data repositories and improve the performance of risk prediction; simultaneously, it also has high accuracy and the ability for estimating the variable importance during classification (17).

Herein, the present study aimed to explore and describe factors related to ND after sICH. Importantly, we established a random forest model in predicting the outcome of ND, with a goal of evaluating the patients’ conditions and identifying those patients at high risk of ND in order to implement early interventions for patients.

## Methods

### Patient Selection

In this case–control study, the clinical data of 413 patients with acute sICH between January 2018 and December 2020 were collected from the Affiliated Hospital of Jining Medical University (*n* = 218) and Xuanwu Hospital Capital Medical University (*n* = 195). Inclusion criteria: (1) patients with age ≥18 years; (2) patients complying with the American Heart Association/American Stroke Association ICH guidelines published in 2010; and (3) patients with complete medical records (including baseline data, laboratory tests, imaging data, treatment records, and prognostic data). Exclusion criteria: (1) patients with ICH caused by craniocerebral trauma, brain tumor, or cerebrovascular malformation; (2) patients taking anticoagulant or antiplatelet drugs before the onset of the illness; (3) patient who died within 7 days of hospitalization; and (4) patients with congenital or acquired coagulation factor deficiency or platelet abnormalities. There are abnormal body temperatures of two patients in the baseline information, 3.6 and 70°C respectively, so these two samples are deleted. Selected patients were divided into the ND group and the non-ND group. This study was approved by the Institutional Review Board of the Affiliated Hospital of Jining Medical University (approval No. 2021C023) and Xuanwu Hospital Capital Medical University (approval No. [2019]085).

### Neurological Deterioration

ND was defined as an increase in the National Institutes of Health Stroke Scale (NIHSS) of ≥4 points or a decline in the Glasgow Coma Scale (GCS) of ≥2 points from onset to 7th day (18). Herein, we measured the NIHSS or GCS for patients at the onset of 24 h and on the 7th day after onset, separately, to identify the patients with ND.

### Variables’ Collection

At admission, data collected included the patients’ age, gender, Body Mass Index (BMI), time of onset to emergency department (ED), SBP on admission, diastolic blood pressure (DBP) on admission, body temperature, baseline hematoma volume, history of high blood pressure (HBP), smoking history, drinking history, other medical history, serum sodium, serum calcium, hemoglobin (Hb), white blood cell (WBC) count, platelet (PLT) count, activated partial thromboplastin time, international normalized ratio (INR), fibrous protein, blood glucose, serum creatinine, troponin, total cholesterol (TCHO), low-density lipoprotein cholesterin (LDL-C), high-density lipoprotein cholesterol (HDL-C), triglyceride (TG), bleeding part, IVH, IVH expansion, subarachnoid expansion, DBP change in 24 h, SBP change in 24 h, hematoma volume change in 24 h, blend sign, spot sign, leukodystrophy, lacuna cerebri, and brain atrophy (any central or cortical reduction). The hematoma volume was calculated according to the Coniglobus formula (19): *V* = *a* × *b* × *c* × 1/2 (where *a* represents the longest diameter of hematoma at the level of maximum hematoma area, *b* represents the longest diameter perpendicular to the longest diameter at the level of maximum hematoma area, and *c* represents the number of layers with bleeding in CT images). The proportion of missing values for most of the included variables was less than 7%, which were filled using multiple imputation. There are 91 (22.14%) missing values in hematoma volume change in 24 h. After filling in the baseline volume of hematoma and the volume of hematoma at 24 h of onset, the volume of hematoma at 24 h minus the volume of baseline hematoma was calculated.

### Development and Validation of the Random Forest Model

The random forest model, as an integrated learning method that combines multiple decision tree, could randomly select the variables in each decision tree as predictors (16). It is worth noting that the random forest model could deal with the problem of certain data loss and provide the important score of each variable (17). In the present study, we made the total samples to randomly split into the training set for the development of the prediction model and the testing set for the internal validation with a ratio of 7:3. In order to improve the generalization ability of the established random forest model, variables that have been identified as contributing to ND in the literature were recruited into the model (20, 21). Herein, we developed a random forest model in predicting the risk of ND after sICH and calculated the importance scores of each variable, which suggested the predictive value of each variable for predicting ND. Additionally, we used the area under (AUC) the receiver operating characteristic (ROC) curve to assess the predicted performance of this random forest model.

### Statistical Analysis

In the present study, we adopted the Shapiro test to test the normality of measurement data. Mean ± standard deviation (Mean ± SD) described the normally distributed measurement data, and differences of the ND group and non-ND group were compared by the *t*-test. The non-normally distributed measurement data were displayed as median and quartiles [M (Q1, Q3)], and the Mann–Whitney *U*-test was used to perform the between-group comparisons. And categorical data were presented by the number of cases and the constituent ratio [*N* (%)], and the *χ*^2^ test was adopted for the comparisons between the ND group and non-ND group.

First, we conducted the descriptive statistics of baseline data and difference analysis between the ND group and the non-ND group. Multivariate logistic analysis was used to investigate the factors related to the ND in patients with acute ICH after adjusting for relevant confounding factors. Then, the random forest model in predicting the risk of ND after sICH was developed in the training set and was validated in the testing set by the ROC curve. Finally, the testing set was divided into five subgroups according to different bleeding sites to verify the prediction results of the prediction model. The odds ratio (OR) and 95% confidence interval (CI) were calculated. The statistical analyses were performed using R (4.0.3) and Python (3.8.3) software. All statistical tests were two-sided, and *p *< 0.05 was considered to be statistically significant.

## Results

### Baseline Characteristics

After deleting two samples with the abnormal body temperatures from Xuanwu Hospital Capital Medical University, a total of 411 patients were finally recruited for this case–control study, who were divided into the ND group (*n* = 178) and the non-ND group (*n* = 233). The characteristics of the ND and non-ND groups are compared in Table 1. The results showed that the median time of onset to ED of the non-ND group was longer than that of the ND group [24 h vs. 8 h, *Z* = 4.412, *p *< 0.001]. The median SBP at admission in the non-ND group was lower than that of patients in the ND group [160.00 mmHg vs. 166.50 mmHg, *Z* = −2.191, *p *= 0.028]. The median baseline hematoma volume of patients in the non-ND group was smaller than that of patients in the ND group [18.00 ml vs. 20.50 ml, *Z* = −2.572, *p *= 0.010]. The average body temperature at admission was lower in the non-ND group [36.61°C vs. 36.76°C, *t* = −2.331, *p *= 0.021]. In laboratory indicators, the serum sodium, the proportion of IVH, the hematoma volume change within 24 h of onset in the non-ND group was lower than that of the ND group. The median serum calcium of the non-ND group was higher than that of the ND group. Detailed information is shown in Table 1.

**Table 1:** Differential analysis of baseline information.

Variables	Total (*n* = 411)	Group		Statistics	*p*
		Non-ND group (*n* = 233)	ND group (*n* = 178)		
Gender, *n* (%)				*χ*^2 ^= 0.258	0.611
Male	268 (65.21)	149 (63.95)	119 (66.85)		
Female	143 (34.79)	84 (36.05)	59 (33.15)		
Age (years), Mean ± SD	59.43 ± 12.60	58.80 ± 12.87	60.25 ± 12.23	*t* = −1.160	0.247
BMI (kg/m^2^), M (Q_1_, Q_3_)	25.04 (22.56, 27.43)	25.25 (22.53, 27.77)	24.80 (22.59, 26.54)	*Z* = 1.678	0.093
Time of onset to ED (h), M (Q_1_, Q_3_)	13.00 (3.00, 48.00)	24.00 (4.00, 48.00)	8.00 (2.00, 24.00)	*Z* = 4.412	<0.001
SBP on admission (mmHg), M (Q_1_, Q_3_)	162.00 (142.50, 183.00)	160.00 (140.00, 180.00)	166.50 (149.25, 187.00)	*Z* = −2.191	0.028
DBP on admission (mmHg), M (Q_1_, Q_3_)	91.00 (80.00, 104.50)	90.00 (80.00, 104.00)	91.50 (80.00, 105.00)	*Z* = −0.799	0.425
Body temperature (°C), Mean ± SD	36.68 ± 0.60	36.61 ± 0.45	36.76 ± 0.75	*t *= −2.331	0.021
Baseline hematoma volume (ml), M (Q_1_, Q_3_)	20.00 (10.00, 35.00)	18.00 (10.00, 30.00)	20.50 (10.00, 50.00)	*Z* = −2.572	0.010
Hypertension, *n* (%)	273 (66.42)	149 (63.95)	124 (69.66)	*χ*^2 ^= 1.232	0.267
Smoking history, *n* (%)	145 (35.28)	81 (34.76)	64 (35.96)	*χ*^2 ^= 0.021	0.884
Drinking history, *n* (%)	151 (36.74)	84 (36.05)	67 (37.64)	*χ*^2 ^= 0.052	0.820
Other medical history, *n* (%)	108 (26.28)	63 (27.04)	45 (25.28)	*χ*^2 ^= 0.083	0.773
Serum sodium (mmol/l), M (Q_1_, Q_3_)	140.00 (137.75, 142.35)	139.80 (137.00, 142.00)	140.30 (138.00, 143.00)	*Z* = −2.664	0.008
Serum calcium (mmol/l), M (Q_1_, Q_3_)	2.18 (2.06, 2.25)	2.21 (2.10, 2.27)	2.13 (2.01, 2.21)	*Z* = 4.444	<0.001
Hemoglobin (g/l), M (Q_1_, Q_3_)	138.00 (124.00, 150.00)	138.00 (126.00, 150.00)	137.50 (120.25, 149.75)	*Z* = 1.006	0.315
WBC count (10^9^/L), M (Q_1_, Q_3_)	8.80 (7.12, 12.00)	8.60 (6.77, 11.30)	8.90 (7.54, 12.37)	*Z* = −2.175	0.030
PLT count (10^9^/L), M (Q_1_, Q_3_)	212.00 (176.50, 249.00)	220.00 (186.00, 252.00)	206.00 (170.25, 246.00)	*Z* = 2.006	0.045
Activated partial thromboplastin time (s), M (Q_1_, Q_3_)	31.60 (27.90, 35.60)	32.10 (28.40, 36.30)	30.95 (27.40, 35.45)	*Z* = 1.607	0.108
INR (s), M (Q_1_, Q_3_)	1.02 (0.97, 1.09)	1.01 (0.96, 1.08)	1.04 (0.97, 1.11)	*Z* = −2.397	0.017
Fibrous protein (g/l), M (Q_1_, Q_3_)	3.30 (2.70, 4.11)	3.40 (2.70, 4.13)	3.20 (2.64, 3.91)	*Z* = 1.370	0.171
Blood glucose (mmol/l), M (Q_1_, Q_3_)	6.40 (5.44, 8.02)	6.20 (5.40, 7.50)	6.60 (5.56, 8.68)	*Z* = −2.377	0.017
Serum creatinine (umol/l), M (Q_1_, Q_3_)	60.40 (51.65, 73.40)	59.80 (51.30, 72.00)	62.20 (52.47, 74.00)	*Z* = −1.460	0.145
Troponin, ug/l, M (Q_1_, Q_3_)	0.12 (0.01, 1.05)	0.67 (0.01, 1.08)	0.08 (0.01, 1.01)	*Z* = 0.048	0.962
TCHO (mg/dl), M (Q_1_, Q_3_)	4.13 (3.50, 4.84)	4.23 (3.56, 4.95)	4.09 (3.42, 4.71)	*Z* = 1.854	0.064
LDL-C (mg/dl), M (Q_1_, Q_3_)	2.51 (1.92, 3.12)	2.60 (2.00, 3.17)	2.46 (1.81, 3.01)	*Z* = 1.906	0.057
HDL-C (mg/dl), M (Q_1_, Q_3_)	1.15 (0.96, 1.36)	1.18 (0.97, 1.38)	1.12 (0.95, 1.32)	*Z* = 1.341	0.180
Triglyceride (mg/dl), M (Q_1_, Q_3_)	1.16 (0.84, 1.61)	1.19 (0.87, 1.63)	1.13 (0.80, 1.61)	*Z* = 1.113	0.266
Bleeding part, *n* (%)				*χ*^2 ^= 4.448	0.349
Others	90 (21.90)	56 (24.03)	34 (19.10)		
Basal ganglia	199 (48.42)	111 (47.64)	88 (49.44)		
Brain stem	25 (6.08)	10 (4.29)	15 (8.43)		
Brain lobe	74 (18.00)	44 (18.88)	30 (16.85)		
Cerebellum	23 (5.60)	12 (5.15)	11 (6.18)		
IVH, *n* (%)	132 (32.12)	61 (26.18)	71 (39.89)	*χ*^2 ^= 8.079	0.004
IVH expansion, *n* (%)	27 (6.57)	17 (7.30)	10 (5.62)	*χ*^2 ^= 0.230	0.632
Subarachnoid expansion, *n* (%)	9 (2.19)	6 (2.58)	3 (1.69)	Fisher	0.738
DBP change in 24 h (mmHg), M (Q_1_, Q_3_)	13.00 (5.00, 27.00)	13.00 (4.00, 25.00)	13.00 (5.00, 28.00)	*Z* = −1.103	0.321
SBP change in 24 h (mmHg), M (Q_1_, Q_3_)	19.00 (3.00, 43.50)	16.00 (2.00, 41.00)	20.50 (5.00, 50.75)	*Z* = −1.667	0.098
Hematoma volume change in 24 h (ml), M (Q_1_, Q_3_)	0.00 (0.00, 15.00)	0.00 (0.00, 10.00)	3.00 (0.00, 20.00)	*Z* = −2.846	0.008
Blend sign, *n* (%)	51 (12.41)	25 (10.73)	26 (14.61)	*χ*^2 ^= 1.062	0.303
Spot sign, *n* (%)	90 (21.90)	48 (20.60)	42 (23.60)	*χ*^2 ^= 0.369	0.544
Leukodystrophy, *n* (%)	105 (25.55)	60 (25.75)	45 (25.28)	*χ*^2 ^= 0.000	1.000
Lacuna cerebri, *n* (%)	139 (33.82)	79 (33.91)	60 (33.71)	*χ*^2 ^= 0.000	1.000
Brain atrophy, *n* (%)	59 (14.36)	24 (10.30)	35 (19.66)	*χ*^2 ^= 6.453	0.011

*ND, neurological deterioration; BMI, body mass index; ED, Emergency Department; SBP, systolic blood pressure; DBP, diastolic blood pressure; IVH, intraventricular hemorrhage; WBC, white blood cell; PLT, platelet; INR, international normalized ratio; TCHO, total cholesterol; LDL-C, low-density lipoprotein cholesterin; HDL-C, high-density lipoprotein cholesterol.*

### The Factors Related to the Neurological Deterioration

**Figure 1:**
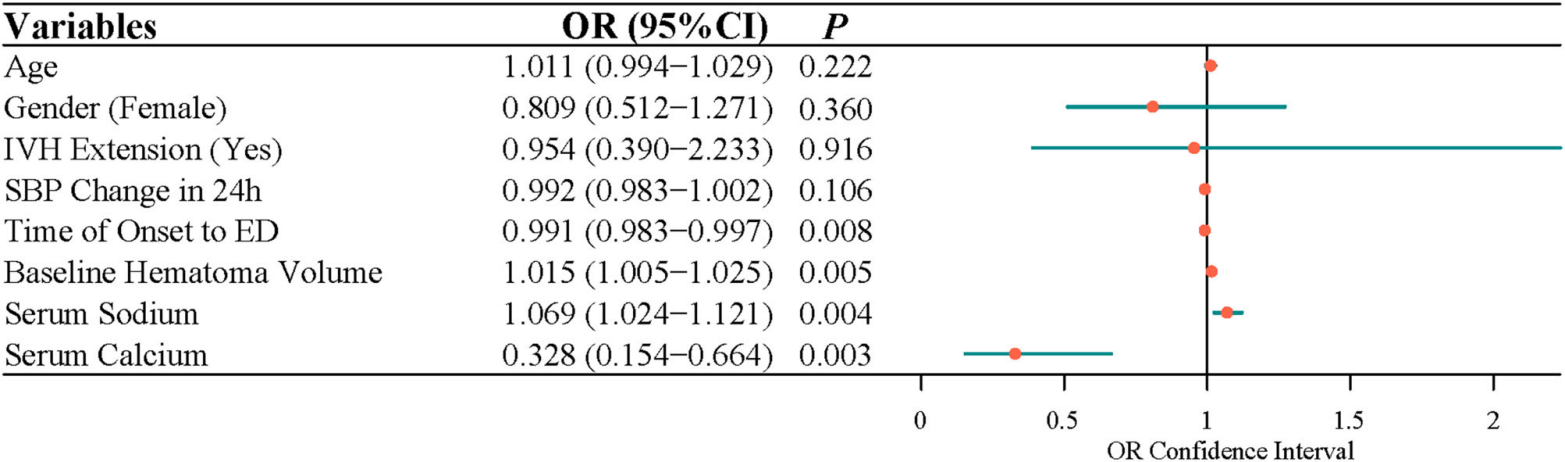
The factors related to the neurological deterioration in patients with acute intracerebral hemorrhage by multivariate logistic analysis.

**Figure 2:**
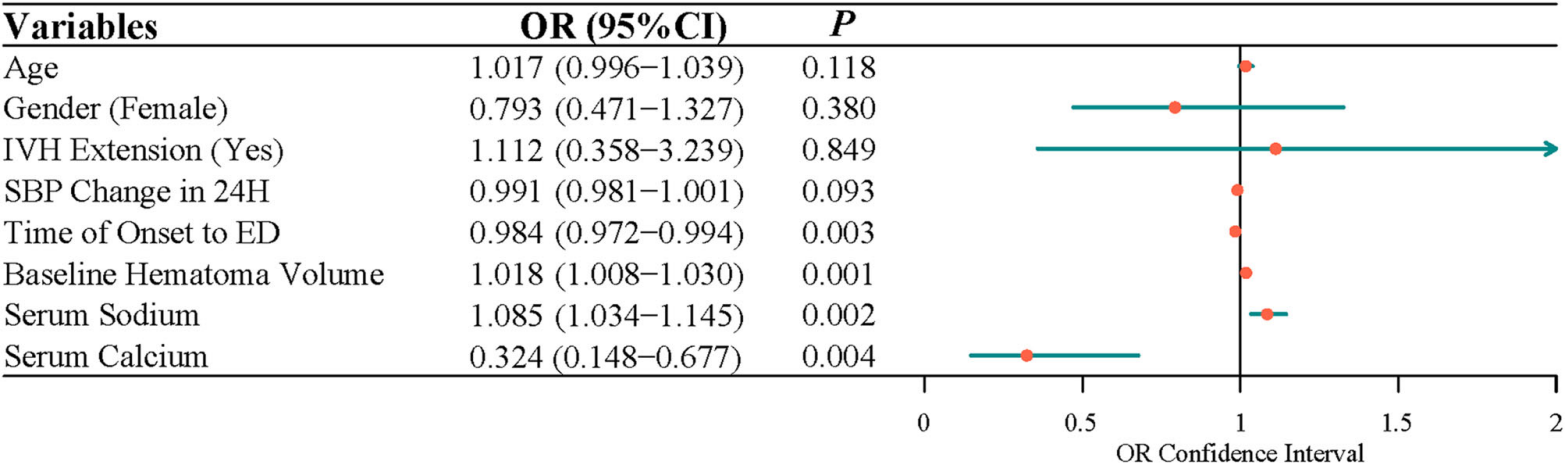
The factors related to the neurological deterioration in patients with acute intracerebral hemorrhage by multivariate logistic analysis (before interpolation).

After adjusting for age, gender, IVH expansion, and SBP change in 24 h, multivariate logistic regression indicated that the time of onset to ED (OR = 0.991, 95% CI, 0.983–0.997), baseline hematoma volume (OR = 1.015, 95% CI, 1.005–1.025), serum sodium (OR = 1.069, 95% CI, 1.024–1.121), and serum calcium (OR = 0.328, 95% CI, 0.154–0.664) were independently associated with the risk of ND (Figure 1). Because data were filled, a sensitivity analysis was carried out, and the conclusion was basically the same as that before the interpolation (Figure 2).

### Establishment and Performance of the Random Forest Model

Not only that, we made these subjects to randomly divide into the training set (*n *= 287) for the development of the prediction model and the testing set (*n *= 124) for the internal validation. As shown in Table 2, no significant differences were displayed between the training set and testing set regarding the baseline characteristics of subjects (*p* > 0.05), which indicated the balance of data division in the training set and testing set.

**Table 2:** Differences between the training set and testing set.

Variables	Total (*n* = 411)	Training set (*n* = 287)	Testing set (*n* = 124)	Statistics	*p*
Onset to ED Arrival (h), M (Q_1_, Q_3_)	13.00 (3.00, 48.00)	13.00 (3.00, 48.00)	15.50 (3.00, 48.00)	*Z* = 0.286	0.776
SBP on admission (mmHg), M (Q_1_, Q_3_)	162.00 (142.50, 183.00)	160.00 (141.50, 183.00)	165.00 (145.75, 180.75)	*Z* = 0.617	0.537
Baseline hematoma volume (ml), M (Q_1_, Q_3_)	20.00 (10.00, 35.00)	20.00 (10.00, 30.00)	20.00 (12.00, 40.00)	*Z* = 1.274	0.203
Serum sodium (mmol/l), M (Q_1_, Q_3_)	140.00 (137.75, 142.35)	140.00 (137.70, 142.05)	140.00 (137.98, 142.85)	*Z* = −0.063	0.950
Serum calcium (mmol/l), M (Q_1_, Q_3_)	2.18 (2.06, 2.25)	2.19 (2.07, 2.25)	2.17 (2.05, 2.25)	*Z* = −0.677	0.498
IVH expansion, *n* (%)	27 (6.57)	16 (5.57)	11 (8.87)	*Z* = 1.237	0.307
Brain atrophy, *n* (%)	59 (14.36)	41 (14.29)	18 (14.52)	*χ*^2 ^= 0.000	1.000
SBP Change in 24 h (mmHg), M (Q1, Q3)	19.00 (3.00, 43.50)	19.00 (2.00, 43.00)	20.00 (5.00, 45.25)	*Z* = 0.775	0.439
Age (years), Mean ± SD	59.43 ± 12.60	59.89 ± 13.88	59.23 ± 12.03	*t* = 0.458	0.647
Gender (female), *n* (%)	143 (34.79)	45 (36.29)	98 (34.15)	*χ*^2 ^= 0.094	0.760

*ED, Emergency Department; SBP, systolic blood pressure; IVH, intraventricular hemorrhage.*

**Figure 3:**
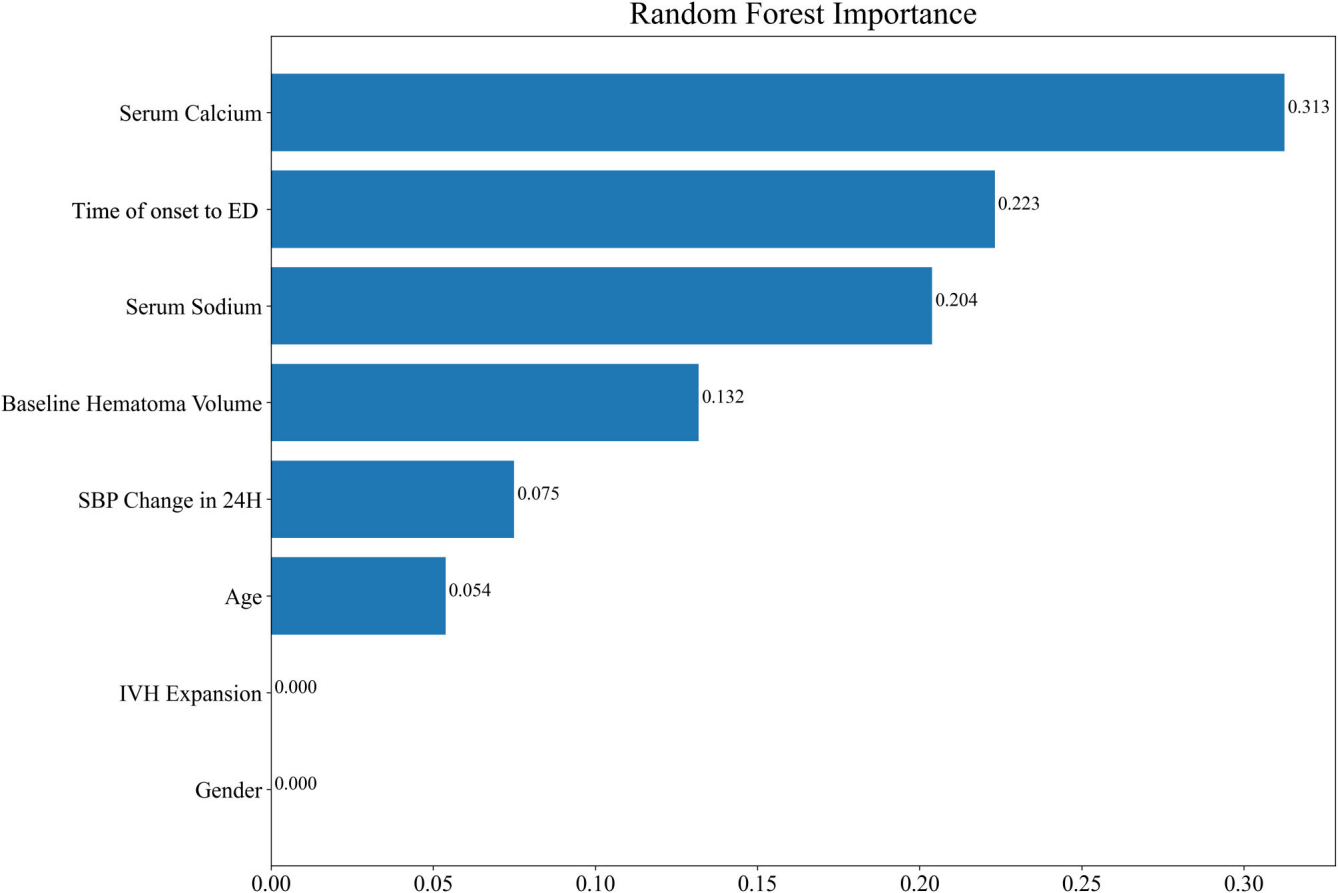
The feature importance diagram of the random forest model.

**Figure 4:**
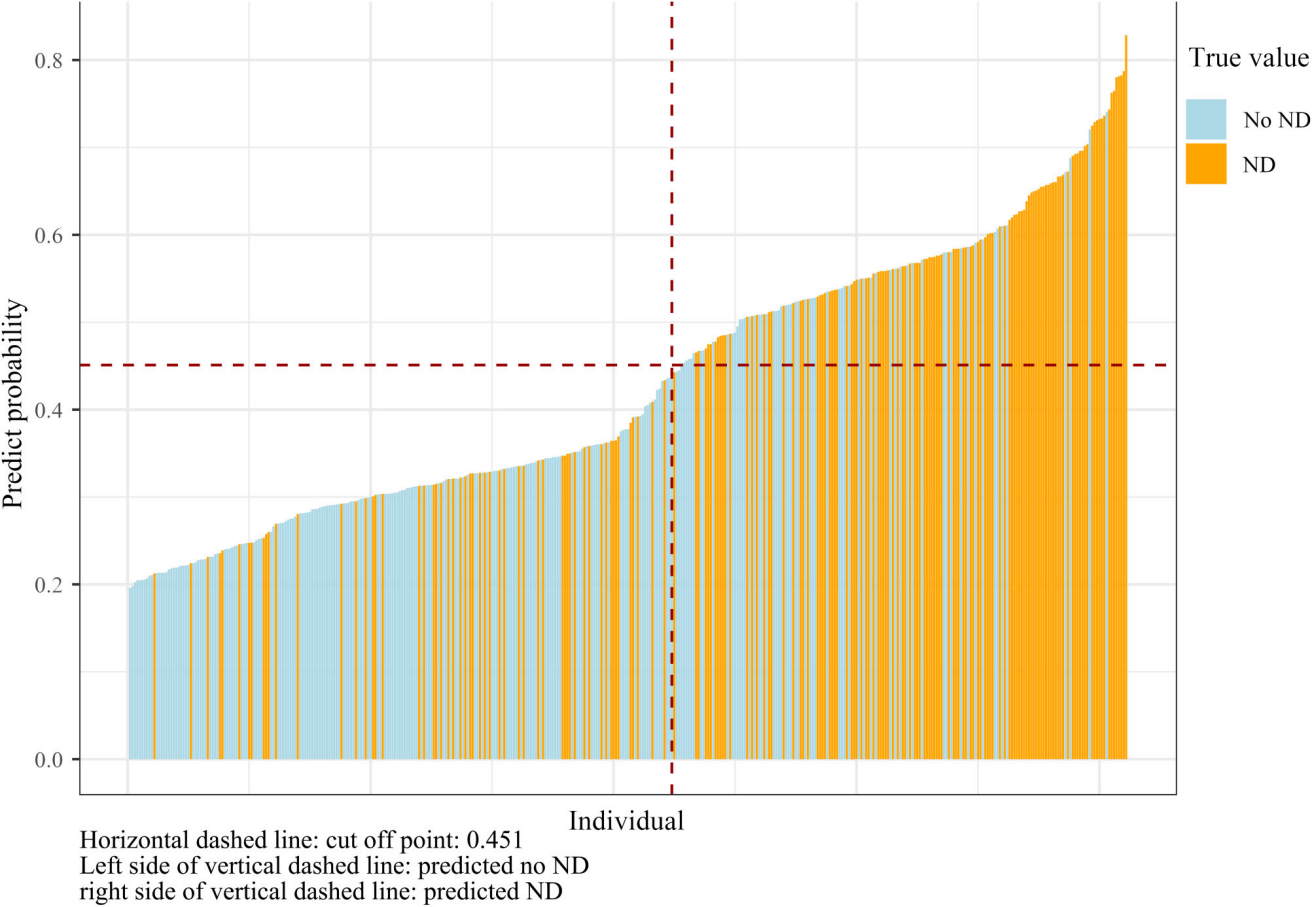
Individual histograms.

In order to improve the generalization ability of the established random forest model, four variables that have been identified as contributing to ND in the literature were recruited into the model (20, 21). Finally, the random forest model was composed of eight factors: serum calcium, the time of onset to ED, serum sodium, baseline hematoma volume, SBP change in 24 h, age, IVH expansion, and gender. The importance scores of the included variables are listed in Figure 3, and the result displayed that serum calcium was the most significant for the risk of ND. Individual histograms showed the predictive effect of the model in the overall data set (Figure 4). The result also suggested that when the predicted probability was lower than 0.451, the individual was considered to have no risk of ND; conversely, the individual has the risk of ND when the predicted probability was higher than 0.451.

Online prediction system: https://github.com/Ischmodel/Ischmodelpredictor.

Table 3 shows the performance of the random forest model in the training set and testing set. The AUC of this model was 0.795, with a sensitivity of 0.737, a specificity of 0.688, a positive predictive value (PPV) of 0.787, and the negative predictive value (NPV) of 0.626 in the training set. The internal validation was conducted to assess the efficacy of the prediction model using the testing set and the results revealed that the AUC was 0.713, and the sensitivity, specificity, PPV, and NPV of the prediction model were 0.697, 0.667, 0.768, and 0.613, respectively (Table 3). The ROC curves of the model are shown in Supplementary Figure 1 (training set) and Supplementary Figure 2 (testing set).

**Table 3:** Prediction effect of the random forest model.

Variables	AUC (95% CI)	Sensitivity (95% CI)	Specificity (95% CI)	PPV (95% CI)	NPV (95% CI)
Training set	0.795 (0.793–0.797)	0.737 (0.686–0.788)	0.688 (0.634–0.741)	0.787 (0.739–0.834)	0.626 (0.570–0.682)
Testing set	0.713 (0.710–0.716)	0.697 (0.617–0.778)	0.667 (0.584–0.750)	0.768 (0.694–0.842)	0.613 (0.495–0.669)

*AUC, area under the curve; CI, confidence interval; PPV, positive predictive value; NPV, negative predictive value.*

The testing set was divided into different subgroups according to different bleeding sites, and the prediction model was verified in the subgroup population. It was found that when the bleeding site was the basal ganglia [AUC (95% CI) = 0.729 (0.724–0.734)], the brain lobe [(AUC (95% CI) = 0.714 (0.707–0.721))] and other bleeding sites [(AUC (95% CI) = 0.791 (0.786–0.797))], the model had a certain predictive ability (Supplementary Figure 3).

## Discussion

In this case–control study, the result indicated that the time of onset to ED, baseline hematoma volume, serum sodium, and serum calcium were independently associated with the risk of ND. Additionally, we also established a random forest model in predicting the risk of ND in patients with acute sICH based on some important factors, including serum calcium, the time of onset to ED, serum sodium, baseline hematoma volume, SBP change in 24 h, age, IVH expansion, and gender. The results showed that the developed random forest model may have a good performance in predicting ND risk of acute ICH patients.

Electrolyte disturbance is a common complication of ICH, which has a close relationship with the prognosis of ICH (22). Previous studies indicated that patients with ICH were prone to have sodium disturbances, which may be related to the syndrome of inappropriate secretion of antidiuretic hormone (SIADH) (22, 23). Sodium disturbances might be associated with perihematomal edema expansion, which caused a poor outcome for patients with ICH (24). Simultaneously, patients with ICH are also prone to have calcium disturbances, which may be associated with coagulation (24). Calcium ion, as a coagulation factor in the human body, is active in most coagulation responses (25). When patients suffered from ICH, cerebral edema, cerebral ischemia, and hypoxia would occur (26). It may cause cell membrane damage, which would lead to a transfer of plentiful calcium ions into cells and a reduction of blood calcium (27). In addition, ICH could trigger a coagulation where calcium ions would be consumed, leading to the development of low serum calcium (28). Our study found that serum sodium and serum calcium was related to the risk of ND. Dastur et al. mentioned in the study on sICH that controls the serum sodium level was beneficial to hematoma expansion reduction (29). A recent study conducted by Mao et al. (27) indicated that the low serum calcium level was associated with a higher risk of HE and poor prognosis after ICH. HE and ND were all common complications of ICH. Leira et al. (30) conducted a multicenter, prospective study to identify factors that predicted or were related to ND in sICH patients, and the results demonstrated that high SBP within 48 h were independent predictors of ND. In this present study, we further investigated SBP change on admission and found that SBP change in 24 h was an important factor for predicting ND. In addition, hematoma volume was also found to be independently correlated with ND (21, 31), which was consistent with our findings.

Another important result of this study was that the establishment of the random forest model to predict the risk of ND after sICH. According to the feature importance scores given in the random forest model, the abscissa was the Gini importance score. The Gini importance score is defined as the Gini impurity difference of the sample set before and after passing a sample feature classification node, that is, the larger the difference, indicated that the higher the score, the more important the variable was (32, 33). Among them, serum calcium was the variable with the highest Gini score. Serum calcium was involved in platelet function during coagulation and plays an important role in cerebral injury after ICH. The serum calcium level was related to impaired hemostasis and HE (27, 29). The overall results of our study demonstrated that serum calcium and serum sodium were important predictors for ND in acute sICH patients. Prior studies tend to establish predictive models using logistic regression analysis and prediction score (21, 31). Miyahara et al. (8) developed a HEAVN prediction score using heterogeneity, peripheral edema, anticoagulant use, volume >30 mL on initial CT, and niveau formation that can be routinely assessed in clinical practice to estimate the probability of HE and ND after sICH. In our study, the AUC value of the prediction model reached 0.795 in the training set and 0.713 in the testing set, which suggested the good predicting value of the model. Not only that, it was found that our model had a certain predictive ability when the bleeding site was the basal ganglia, lobes, and other bleeding sites. Moreover, the AUC of the basal ganglia subgroup, lobes subgroup, and other bleeding sites subgroup was 0.729, 0.714, and 0.791, respectively, indicating the effectiveness of the random forest model in clinical application.

In this study, four influencing factors obtained in logistic regression analysis and four variables that have been identified as contributing to ND in the literature were used to establish the random forest model, which may improve the generalization ability of the established random forest model. Moreover, the testing set was divided into different subgroups according to different bleeding sites, and the prediction model was verified in the subgroups. The effectiveness of the random forest model in the clinical application was demonstrated. There were some limitations in our study. First, inadequate population diversity may cause a poor efficacy of the established model when used in other populations. Second, we excluded some patients who died within 7 days of hospitalization; therefore, the established model only might apply to patients who will survive for more than 7 days. These should be cautious in interpreting the results. Future studies will further include more patients with acute sICH who came from different centers in China, to evaluate the predictive value of the developed model.

## Conclusion

In short, time of onset to ED, baseline hematoma volume, serum sodium, and serum calcium were associated with the risk of ND. Additionally, a prediction model for ND of acute sICH patients was developed based on random forest analysis, and the developed model may have a good predictive value through the internal validation. We believed that the developed random forest model may act as a simple tool to evaluate the population at high risk of exacerbation of ND symptoms, thereby helping clinicians to identify who would need early intervention.

## Data Availability

The raw data supporting the conclusions of this article will be made available by the authors, without undue reservation.

## Supplementary Material

**Supplementary Figure 1 |** The receiver operating characteristic curves for the testing set of the random forest model.

**Supplementary Figure 2 |** The receiver operating characteristic curves for the training set of the random forest model.

**Supplementary Figure 3 |** The receiver operating characteristic curves of the subgroup analyses.
