# Supplementary figures and images for: A Prediction Model for Neurological Deterioration in Patients with Acute Spontaneous Intracerebral Hemorrhage
Source: Front Surg. 2022 May 27;9:886856. doi: 10.3389/fsurg.2022.886856 (PMC9198834; doi:10.3389/fsurg.2022.886856)

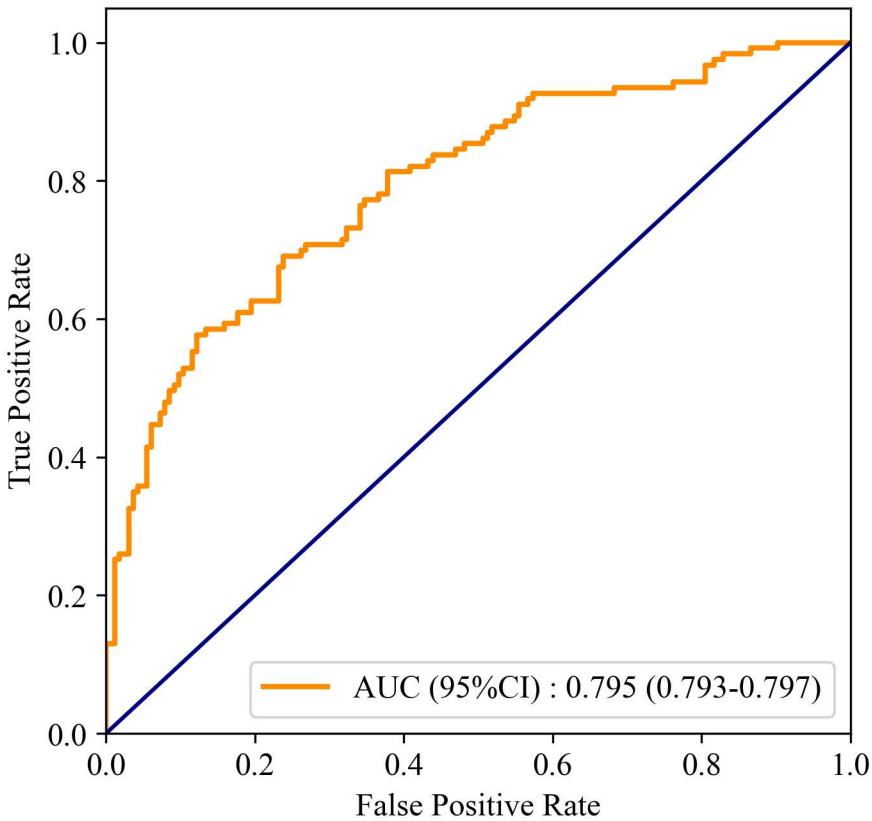

Supplement: Supplementary Figure 1 | The receiver operating characteristic curves for the testing set of the random forest model. [file Data_Sheet_1_v1.pdf]

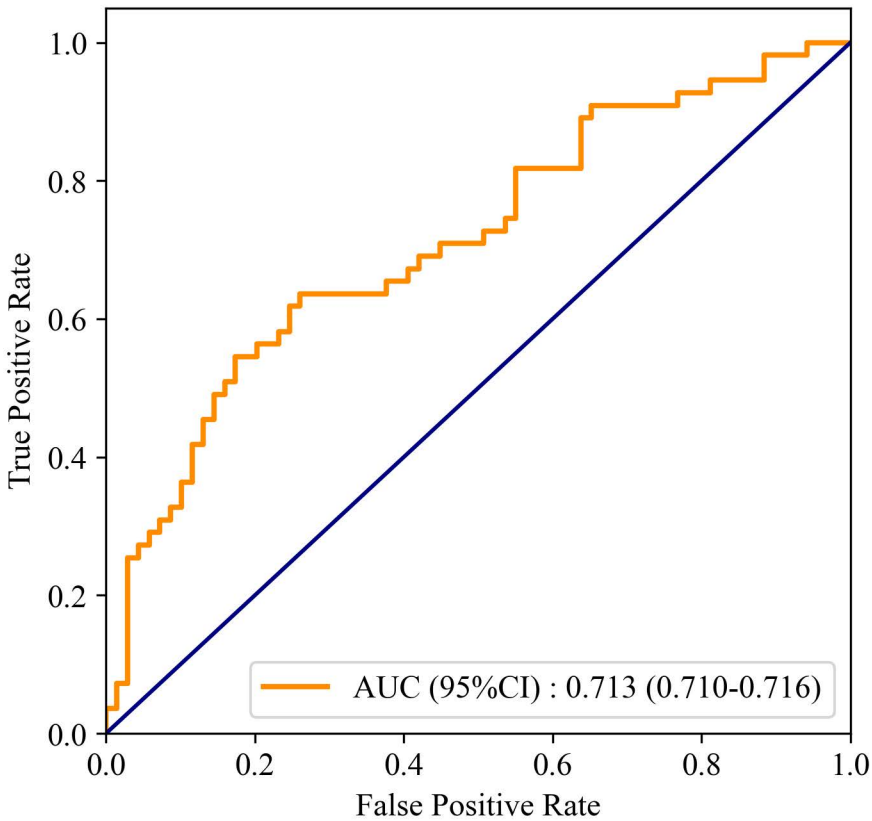

Supplement: Supplementary Figure 2 | The receiver operating characteristic curves for the training set of the random forest model. [file Data_Sheet_2_v1.pdf]
