# Supplementary material for: A Prediction Model for Neurological Deterioration in Patients with Acute Spontaneous Intracerebral Hemorrhage
Source: Front Surg. 2022 May 27;9:886856. doi: 10.3389/fsurg.2022.886856 (PMC9198834; doi:10.3389/fsurg.2022.886856)
Supplement: Supplementary Figure 3 | The receiver operating characteristic curves of the subgroup analyses. [file Data_Sheet_3_v1.pdf]

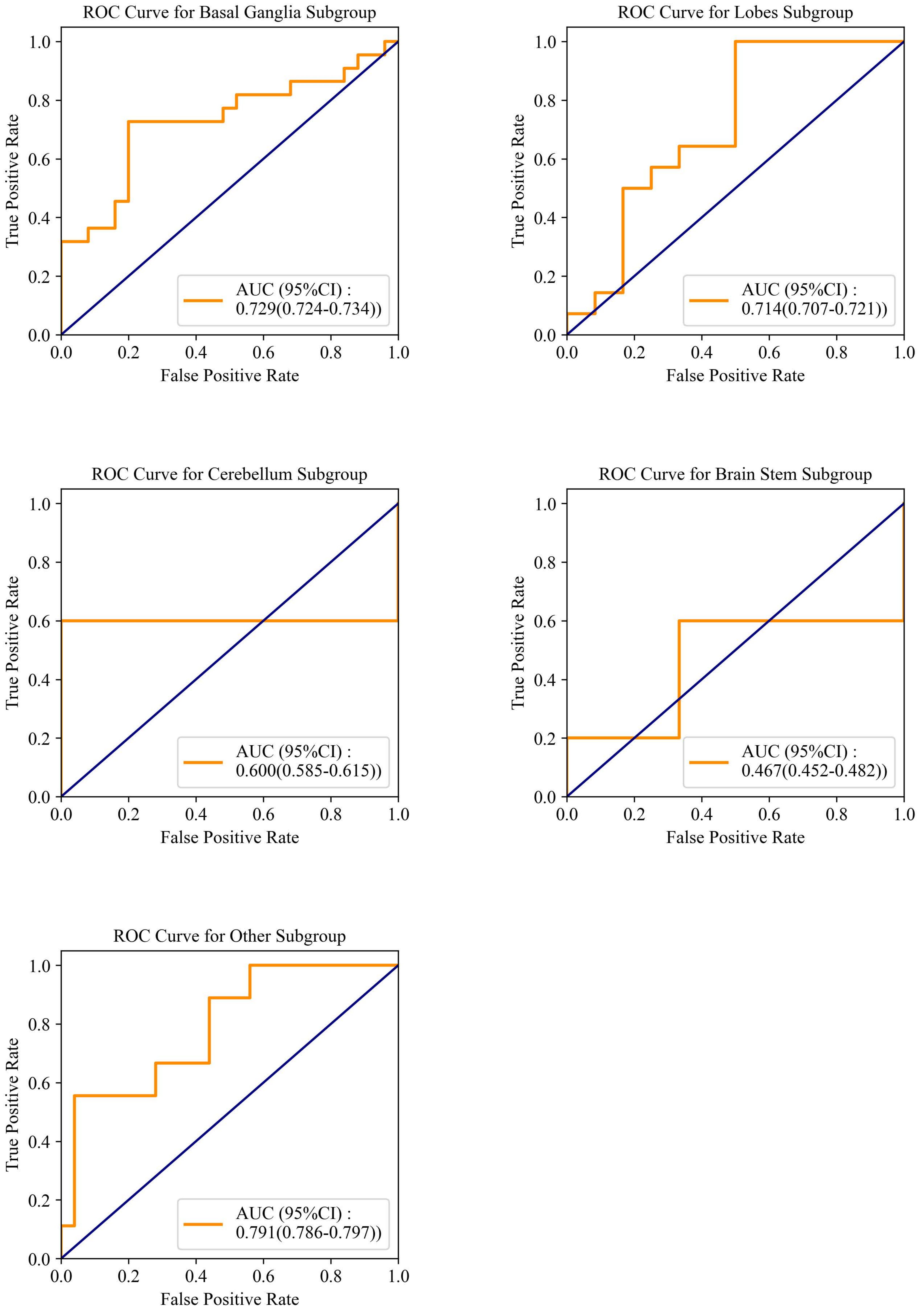
ROC Curve for Basal Ganglia Subgroup
True Positive Rate
False Positive Rate
0.0
0.2
0.4
0.6
0.8
1.0
AUC (95%CI) :
0.729(0.724-0.734))
ROC Curve for Lobes Subgroup
True Positive Rate
False Positive Rate
AUC (95%CI) :
0.714(0.707-0.721))
ROC Curve for Cerebellum Subgroup
True Positive Rate
False Positive Rate
AUC (95%CI) :
0.600(0.585-0.615))
ROC Curve for Brain Stem Subgroup
True Positive Rate
False Positive Rate
AUC (95%CI) :
0.467(0.452-0.482))
ROC Curve for Other Subgroup
True Positive Rate
False Positive Rate
AUC (95%CI) :
0.791(0.786-0.797))
