# Supplementary material for: A Prediction Model for Neurological Deterioration in Patients with Acute Spontaneous Intracerebral Hemorrhage
Source: Front Surg. 2022 May 27;9:886856. doi: 10.3389/fsurg.2022.886856 (PMC9198834; doi:10.3389/fsurg.2022.886856)
Supplement: Supplementary file 4 [file Data_Sheet_4_v1.docx]

**Response Letter**

Dear Editor,

Thank you very much for your letter and advice. We would like to express our sincere thanks to the reviewers for the constructive and positive comments. We have addressed the comments raised by the editor and reviewers, and would like to re-submit it for your consideration. The amendments are highlighted in red in the revised manuscript. Point by point responses to the editor’ and reviewers’ comments are listed below this letter.

We hope that the revised version of the manuscript is now acceptable for publication in your journal.

Look forward to hearing from you soon.

With kind regards,

Yours sincerely,

Yuanyuan Qiao

 Reviewer 1

Thank you for this interesting analysis. Particularly the role of serum calcium and sodium in ICH patients brings some important insights.
Why did you exclude patients taking antithrombotic medication and patients who died within 7 days of hospitalization? Please include a brief explanation.

Response: Thanks very much for your advice. In this study, we excluded some patients who took anticoagulant or antiplatelet drugs before the onset of the illness, or died within 7 days of hospitalization. Several studies have found that there were some differences in the using of anticoagulant drugs between neurological deterioration (ND) group and non-ND group for patients with spontaneous intracerebral hemorrhage [1-2]. In the study of Lord, et al [2]. they explored the time course and predictors of ND after intracerebral hemorrhage, and population who had the use of anticoagulants was excluded. Therefore, we speculated that the use of antithrombotic drugs or anticoagulants might have an impact on the prognosis of patients with spontaneous intracerebral hemorrhage. In order to control the influence of confounding factors, we excluded some patients who took anticoagulant or antiplatelet drugs before the onset of the illness.

In addition, ND was defined as from onset to 7th day, an increase in the National Institutes of Health Stroke Scale (NIHSS) of ≥ 4 points or a decline in the Glasgow Coma Scale (GCS) of ≥ 2 points in this study. Because patients who died within 7 days could not be determined whether ND has occurred, we need exclude these patients who died within 7 days of hospitalization.

[1] You S., Zheng D., Delcourt C., Sato S., Cao Y., Zhang S., et al. (2019). Determinants of Early Versus Delayed Neurological Deterioration in Intracerebral Hemorrhage. *Stroke* 50(6), 1409-1414. doi: 10.1161/STROKEAHA.118.024403.

[2] Lord AS., Gilmore E., Choi HA., Mayer SA., VISTA-ICH Collaboration., et al. (2015). Time course and predictors of neurological deterioration after intracerebral hemorrhage. *Stroke* 46(3), 647-52. doi: 10.1161/STROKEAHA.114.007704.

Can you discuss the pathophysiology of calcium and sodium disturbances in ICH patients? Do we know why patients can develop low serum calcium in the context of ICH? Do you recommend to substitute calcium in the acute setting of ICH?
Response: Thanks very much for your opinion. Electrolyte disturbance is a common complication of ICH, which has a close relationship with the prognosis of ICH [1]. Previous studies indicated that patients with ICH were prone to have sodium disturbances, which may be related to the syndrome of inappropriate secretion of antidiuretic hormone (SIADH) [1-2]. Sodium disturbances might be associated with perihematomal edema expansion, which caused a poor outcome for patients with ICH [3]. Simultaneously, patients with ICH are also prone to have calcium disturbances, which may be associated with coagulation [3]. Calcium ion, as a coagulation factor in human body, is active in most coagulation responses [4]. When patients suffered from ICH, cerebral edema, cerebral ischemia and hypoxia would occur [5]. It may cause cell membrane damage, which would lead in a transfer of plentiful calcium ions into cells and a reduction of blood calcium [6]. In addition, ICH could trigger a coagulation where calcium ions would be consumed, leading to the development of low serum calcium [7]. The possible reasons of low serum calcium for patients with ICH are as described above. Therefore, our recommendation is to appropriately replenish the calcium in the acute setting of ICH [3].

[1] Liu J., Li Q., Ren J., Liang X., Zhang Q., Han Y. (2020). Association of Sex with Serum Potassium, Sodium, and Calcium Disorders after Hypertensive Intracerebral Hemorrhage. *World Neurosurg* 141, e367-e373. doi: 10.1016/j.wneu.2020.05.137.

[2] Soudan K, Qunibi W. (2012). Severe hypernatremia following treatment of the syndrome of inappropriate antidiuretic hormone secretion. *Am J Med Sci* 343(6), 507-9. doi: 10.1097/MAJ.0b013e318245faaf.

[3] Loggini A., El Ammar F., Mansour A., Kramer CL., Goldenberg FD., Lazaridis C. (2021). Association between electrolyte levels at presentation and hematoma expansion and outcome in spontaneous intracerebral hemorrhage: A systematic review. *J Crit Care* 61, 177-185. doi: 10.1016/j.jcrc.2020.10.029.

[4] Palta S., Saroa R., Palta A. (2014). Overview of the coagulation system. *Indian J Anaesth* 58(5), 515-23

[5] Hemphill JC., Greenberg SM., Anderson CS., Becker K., Bendok BR., Cushman M., et al. (2015). Guidelines for the Management of Spontaneous Intracerebral Hemorrhage: A Guideline for Healthcare Professionals from the American Heart Association/American Stroke Association. *Stroke* 46(7), 2032-60.

[6] Mao J., Jiang W., Liu G., Jiang B. (2019). Serum calcium levels at admission is associated with the outcomes in patients with hypertensive intracerebral hemorrhage. *Br J Neurosurg* 33(2), 145-148.

[7] Sun J., Liu W., Zhu R., Wu Y., Yang L. (2020). The relationship between low serum calcium level and intracerebral hemorrhage hematoma expansion: A protocol of systematic review and meta-analysis. *Medicine (Baltimore)* 99(3), e18844.

Has your statistical analysis been reviewed by a biomedical statistician?

Response: Thanks very much for your opinion. The statistical analysis of this manuscript has been reviewed by a biomedical statistician. Please see the revised manuscript.

In the abstract: please write out the abbreviation “ED”.
Response: Thanks very much for your advice. We have added the content in the abstract: emergency department (ED).

Personally, I would remove “two hospitals” in the title of the paper. It is described in the Methods section.

Response: Thanks very much for your advice. According to your comments, we have removed “two hospitals” in the title of manuscript.

a. Is the quality of the figures and tables satisfactory?
- Yes
Response: Thanks for your suggestion.

b. Does the reference list cover the relevant literature adequately and in an unbiased manner?
- Yes

Response: Thanks very much for your advice.

c. Are the statistical methods valid and correctly applied? (e.g. sample size, choice of test)
- Yes
Response: Thanks very much for your advice.

d. Is a statistician required to evaluate this study?
- Yes
Response: Thanks for your suggestion.

e. Are the methods sufficiently documented to allow replication studies?
- Yes

Response: Thanks very much for your opinion.

Reviewer 2

This is a retrospective series, of a reasonable size (411 patients) in China who suffered acute ICH, attempting to create a model to predict neurological deterioration. The authors found that time from presentation, hematoma volume, serium Ca and Na were associated with an increased risk of deterioration. Their forest plot identified a few different factors that were predictive in their model.
The manuscript is well written and the data is appropriately analyzed and reported. Some minor grammatical mistakes could be corrected.
Response: Thanks for your suggestion. We have carefully reedited language for the entire manuscript. Please see the revised manuscript.

This is not a novel study, as the authors identify, there have been multiple other studies attempting to identify factors associated with ND after ICH. In that sense, this paper is additive rather than novel. However, I do believe it has some value as each of these types of paper tends to add clarity around risk factors, and their predictive model is a new addition.
Response: Thanks very much for your advice. In this study, we explored risk factors related to neurological deterioration (ND) after spontaneous intracerebral hemorrhage (sICH). The result showed that time of onset to emergency department (ED), baseline hematoma volume, serum sodium, and serum calcium were independently associated with the risk of ND. Importantly, we established a random forest model based on serum calcium, the time of onset to ED, serum sodium, baseline hematoma volume, SBP change in 24h, age, IVH expansion and gender in predicting the outcome of ND for acute sICH patients. The developed model may have a good predictive value through the internal validation. We believed that the developed random forest model may act as a simple tool to evaluate the population at high risk of exacerbation of ND symptoms, thereby helping clinicians to identify who would need early intervention.

An AUC of <0.8 indicates a fair correlation in clinical practice, as you would hope for even >0.9 to indicate an excellent correlation. In practice, often AUC <0.8 may still provide some value but the strength of their conclusions should be tempered by this.

Response: Thanks for your suggestion. According to your comments, we have modified the conclusion of manuscript as following: Additionally, a prediction model for ND of acute sICH patients was developed based on random forest analysis, and the developed model may have a good predictive value through the internal validation. Please see the revised manuscript.

The authors should explain some of the more technical terminology for a clinical audience – e.g. Gini importance score, HEAVN prediction score.

Response: Thanks very much for your advice. Gini importance score is calculated from random forest structure. In decision tree and random forest algorithms, Gini importance is defined as the Gini impurity difference of the sample set before and after passing a sample feature classification node, that is, the larger the difference, the more important the sample feature is in classification [1-2]. HEAVN prediction score was developed by Miyahara et al. to estimate the probability of hematoma expansion and neurological deterioration after sICH [3]. The HEAVN prediction score was composed of five variables, including heterogeneity, peripheral edema, anticoagulant use, volume >30 mL on initial CT and niveau formation. We have added explanation of Gini importance score and HEAVN prediction score in the revised manuscript.
[1] Menze BH., Kelm BM., Masuch R., Himmelreich U., Bachert P., Petrich W., et al. (2009). A comparison of random forest and its Gini importance with standard chemometric methods for the feature selection and classification of spectral data. *BMC Bioinformatics* 10, 213. doi: 10.1186/1471-2105-10-213.

[2] Liu Y., Guo Y., Wu W., Xiong Y., Sun C., Yuan L., et al. (2019). A Machine Learning-Based QSAR Model for Benzimidazole Derivatives as Corrosion Inhibitors by Incorporating Comprehensive Feature Selection. *Interdiscip Sci* 11(4), 738-747. doi: 10.1007/s12539-019-00346-7.

[3] Miyahara, M., Noda, R., Yamaguchi, S., Tamai, Y., Inoue, M., Okamoto, K., et al. (2018). New Prediction Score for Hematoma Expansion and Neurological Deterioration after Spontaneous Intracerebral Hemorrhage: A Hospital-Based Retrospective Cohort Study. *J Stroke Cerebrovasc Dis* 27(9), 2543-2550. doi: 10.1016/j.jstrokecerebrovasdis.2018.05.018.

The authors should next attempt to validate their model with an external data set and see if it is of a similar predictive value.

Response: Thanks for your suggestion. In this study, a random forest model was developed to predict the risk of ND among patients with acute sICH who came from the Affiliated Hospital of Jining Medical University and Xuanwu Hospital of Capital Medical University. The result also indicated that the developed model may have a good predictive value. In the future, we will consider to include more patients with acute sICH who came from different centers in China, to evaluate the predictive value of the developed model.

a. Is the quality of the figures and tables satisfactory?
- Yes
Response: Thanks for your suggestion.

b. Does the reference list cover the relevant literature adequately and in an unbiased manner?
- Yes
Response: Thanks very much for your advice.

c. Are the statistical methods valid and correctly applied? (e.g. sample size, choice of test)
- Yes
Response: Thanks very much for your advice.

d. Is a statistician required to evaluate this study?
- No
Response: Thanks very much for your opinion.

e. Are the methods sufficiently documented to allow replication studies?
- Yes

Response: Thanks for your suggestion.

**Reviewer 3**

For ND, when was the measurement being taken? How to measure the haematoma volume?

Response: Thanks for your suggestion. In our study, neurological deterioration (ND) was defined as an increase in the National Institutes of Health Stroke Scale (NIHSS) of ≥ 4 points or a decline in the Glasgow Coma Scale (GCS) of ≥ 2 points from onset to day 7 [1]. Herein, we measured the NIHSS or GCS for all patients at the onset of 24h and at 7th day after onset, separately., to identify the patients with ND. The hematoma volume was calculated by the Coniglobus formula [2]: *V = a × b × c ×* 1/2, (*a* represents the longest diameter of hematoma at the level of maximum hematoma area; *b* represents the longest diameter perpendicular to the longest diameter at the level of maximum hematoma area; *c* represents the number of layers with bleeding in CT images). We have added these contents in methods section. Please see the revised manuscript.

[1] Morotti, A., Charidimou, A., Phuah, C.L., Jessel, M.J., Schwab, K., Ayres, A.M., et al. (2016). Association Between Serum Calcium Level and Extent of Bleeding in Patients with Intracerebral Hemorrhage. *JAMA Neurol* 73(11), 1285-1290. doi: 10.1001/jamaneurol.2016.2252.

[2] Gao Y., Fu X., Yu L., Zhang D., Lu Z., Cui K., et al. (2021). DNA Hypomethylation of DOCK_1_ Leading to High Expression Correlates with Neurologic Deterioration and Poor Function Outcomes after Spontaneous Intracerebral Hemorrhage. *Evid Based Complement Alternat Med* 1186458. doi: 10.1155/2021/1186458.

In multivariate logistic regression, only four factors were identified to be independently associated with the risk of ND. But in establishing random forest model, another four factors were recruited based on literatures, which, seems to disregard the findings of logistic regression.

Response: Thanks for your suggestion. In this study, the result of multivariate logistic regression showed that time of onset to emergency department (ED), baseline hematoma volume, serum sodium, and serum calcium were independently associated with the risk of neurological deterioration (ND). In order to improve the generalization ability of the established random forest model, four variables that have been identified as contributing to ND in the literature were recruited into model [1-2]. Finally, the random forest model was composed of 8 factors: serum calcium, the time of onset to ED, serum sodium, baseline hematoma volume, SBP change in 24h, age, IVH expansion and gender.

[1] Rodriguez-Luna D., Piñeiro S., Rubiera M., Ribo M., Coscojuela P., Pagola J., et al. (2013). Impact of blood pressure changes and course on hematoma growth in acute intracerebral hemorrhage. *Eur J Neurol* 20(9), 1277-83. doi: 10.1111/ene.12180.

[2] Law ZK., Dineen R., England TJ., Cala L., Mistri AK., Appleton JP., et al. (2021). Predictors and Outcomes of Neurological Deterioration in Intracerebral Hemorrhage: Results from the TICH-2 Randomized Controlled Trial. *Transl Stroke Res* 12(2), 275-283. doi: 10.1007/s12975-020-00845-6.

Patients died within seven days of hospitalization were excluded. But they were the group of patients with the most significant change in condition which included ND, excluding them may exclude the most important factors in the analysis. This also make the model only applied to patients who will survive for more than seven days, which contribute to a major weakness of the model and calculator. Authors need a major revision or mention this in the limitation of study.

Response: Thanks very much for your opinion. Due to the definition of neurological deterioration (ND), we excluded some patients who died within 7 days of hospitalization. However, as you described, these excluded patients might be the group of patients with the most significant change in condition, which contribute to a major weakness of the model. Herein, we already described the limitation in discussion section as following: Second, we excluded some patients who died within 7 days of hospitalization; therefore, the established model only might apply to patients who will survive for more than seven days. These should be cautious in interpreting the results. Please see the revised manuscript.

a. Is the quality of the figures and tables satisfactory?
   - Yes
   Response: Thanks for your suggestion.

b. Does the reference list cover the relevant literature adequately and in an unbiased manner?
- Yes

Response: Thanks very much for your opinion.

c. Are the statistical methods valid and correctly applied? (e.g. sample size, choice of test)
- Yes
Response: Thanks for your suggestion.

d. Is a statistician required to evaluate this study?
- Yes
Response: Thanks very much for your opinion.

e. Are the methods sufficiently documented to allow replication studies?
- Yes

Response: Thanks for your suggestion.
